# Multi-Response Optimization of Electrothermal Micromirror Using Desirability Function-Based Response Surface Methodology

**DOI:** 10.3390/mi8040107

**Published:** 2017-04-01

**Authors:** Muhammad Mubasher Saleem, Umar Farooq, Umer Izhar, Umar Shahbaz Khan

**Affiliations:** 1 Department of Mechatronics Engineering, National University of Sciences and Technology, Islamabad 44000, Pakistan; umerfarooq.nust@gmail.com (U.F.); u.shahbaz@ceme.nust.edu.pk (U.S.K.); 2 School of Engineering and Technology, Central Queensland University, Mackay Ooralea 4740, Australia; u.izhar@cqu.edu.au

**Keywords:** micromirror, Micro-Electro-Mechanical Systems (MEMS), bimorph, optimization, biomedical, desirability function, response surface models

## Abstract

The design of a micromirror for biomedical applications requires multiple output responses to be optimized, given a set of performance parameters and constraints. This paper presents the parametric design optimization of an electrothermally actuated micromirror for the deflection angle, input power, and micromirror temperature rise from the ambient for Optical Coherence Tomography (OCT) system. Initially, a screening design matrix based on the Design of Experiments (DOE) technique is developed and the corresponding output responses are obtained using coupled structural-thermal-electric Finite Element Modeling (FEM). The interaction between the significant design factors is analyzed by developing Response Surface Models (RSM) for the output responses. The output responses are optimized by combining the individual responses into a composite function using desirability function approach. A downhill simplex method, based on the heuristic search algorithm, is implemented on the RSM models to find the optimal levels of the design factors. The predicted values of output responses obtained using multi-response optimization are verified by the FEM simulations.

## 1. Introduction

Micro-Electro-Mechanical Systems (MEMS) technology-based micromirrors are microscale devices used in optical systems to project light over a wide range of reflection angles. Micromirrors are generally used in various applications depending upon their geometric configuration, actuation mechanism, and output performance characteristics. The major application areas of micromirrors include optical switches [1], optical communications [2], optical displays [3,4], microscopic topometry [5], barcode scanning [6], biomedical imaging [7,8], and optical interconnects [9]. The deflection angle of a micromirror can be adjusted statically or dynamically by an actuation mechanism that allows the rotation of the mirror surface. The actuation mechanisms for the deflection of micromirror plates for optical scanning are mainly divided into four categories: electrostatic, piezoelectric, electromagnetic, and electrothermal. The choice of an actuation mechanism is generally dependent on the maximum angular displacement, device size, input power, input voltage, and microfabrication process [10].

Electrothermal actuation for micromirrors allows us to achieve relatively large angular deflection in the mirror plate at low actuation voltages. Moreover, the electrothermal micromirrors have almost linear response between the deflection angle and actuation voltage, high fill factor, simple design, and easy fabrication process. These characteristics make electrothermal micromirrors a suitable choice for biomedical imaging applications [11,12,13,14]. The electrothermal actuators may be designed using either a single thin-film metal structural layer to achieve an in-plane or out-of-plane deflection corresponding to an applied voltage [15,16] or a combination of two material layers (typically a metal and dielectric) bonded at an interface with a significant difference in their coefficients of thermal expansion (CTE) [17,18,19]. When an increase in temperature is applied, the thermal bi-layer actuator bends towards the side of the material that has a lower CTE value. The displacement caused by bending is used in micromirror designs to rotate the mirror surface. A mirror plate is usually attached to the end of the actuator and deflects at an angle equal to the tangential angle of the bimorph end. In optical imaging applications, like an Optical Coherence Tomography (OCT) system, the optical scanning angle has twice the mechanical deflection angle of the mirror plate. Earlier work on the application of the bimorph thermal actuators for the micromirror was presented by Bulher et al. [20]. Ataka et al. [21] reported a bimorph actuator based on a dual-layer polyimide material for the distributed micromotion systems. Yang et al. [22] reported a precise position tracking based on SiO_2_/doped silicon bimorph actuator, where the proposed micromirror can be vertically actuated by 1 µm at an input power of 3 mW. Jain et al. [23] demonstrated an electrothermally actuated micromirror design with optical scan angles larger than ±30° in two dimensions with driving voltages of less than 12 V. The bimorph layers used in this design were aluminum and silicon dioxide. Singh et al. [24] demonstrated an electrothermal micromirror based on an aluminum/silicon bimorph actuator with a reflecting metal-coated silicon mirror plate. The device size was 2.5 mm × 2.5 mm and achieved 17° angular mechanical deflection at an actuation voltage of 1.6 V. Xie et al. [25] presented a micromirror design using Al/SiO_2_ bimorph thermal actuators for laser beam scanning in an OCT system. Izhar et al. [26] presented an electrothermally actuated multi-axis micromirror design for OCT systems and reported an optical scanning angle of 32° with an applied voltage of 6 V and input power of 12 mW. Liu et al. [27] presented a micromirror with aluminum/tungsten bimorphs for fast thermal response with an optical scanning angle of ±11° at 0.6 V. A large optical scanning angle of ±60.4° is reported in [28], at an actuation voltage of 9.8 V, corresponding to a mechanical deflection angle of 18.1° only. The large optical scanning angle is achieved by submerging the mirror into a mineral oil with a refractive index of 1.47 and utilizing the “Snell’s window effect”. Samuelson et al. [29] reported a micromirror actuated by ladder actuators showing 0.25° lateral mechanical deflection angle at 90 μm piston mode displacement, with an actuation voltage of 1.2 V. Jang et al. [30] reported a MEMS-based parallel plate-rotation (PPR) device for a single-imager-based stereoscopic endoscope. The fabricated MEMS PPR device rotates an optical plate with a rotation angle up to 37°. Recently, Duan et al. [31] presented a microendoscopic OCT probe with a tilted electrothermal micromirror, directly integrated on a silicon optical bench. The micromirror consists of a two axis scanning single-crystal-silicon (SCS) mirror tilted using an Al/SiO_2_ bimorph thermal actuator. The maximum scan angle of the mirror plate is 40° at an actuation voltage of 5.5 V for both axes. The main performance characteristics of a micromirror design discussed in the literature for biomedical applications in general, and for an OCT system specifically, include micromirror plate deflection angle and input power. For an OCT system, a higher micromirror deflection angle allows us to scan a large area from a certain distance. The power dissipated in the micromirror due to the electrothermal actuation results in a temperature rise in the device, which adversely affects the output power of the laser integrated in the OCT system [26].

The main challenge in the design of a MEMS device is to obtain the optimal geometric configuration of the device while considering multiple performance constraints. Conventionally, optimization of MEMS devices is carried out by developing analytical models, FEM models, topology optimization, artificial neural networks, and genetic algorithms. These techniques for multiple output responses become impractical due to the complex geometry and high computational costs involved, especially for electrothermal micromirrors, which involve complex structural-thermal-electric interactions. A multi-response optimization using Design of Experiments (DOE) allows for investigating the design space of a MEMS device at different sample points using FEM models with less time, effort, and computational costs and facilitates the analysis of the effect of different parameters on output responses in detail. Previously, authors have discussed the application of the DOE technique for single-response optimization of a RF-MEMS switch to achieve a reliable and optimized design considering the microfabrication process uncertainties and residual stresses [32]. In this paper, a DOE-technique-based multi-response optimization for the scanning electrothermal micromirror design, to be integrated in the sample arm of an OCT system, is presented, considering mirror plate optical deflection angle, input power, and temperature rise from the ambient in the mirror plate.

## 2. Design and Working Principle of the Proposed Micromirror

**Figure 1:**
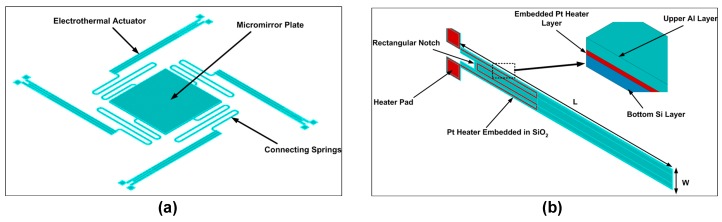
(**a**) Schematic of the micromirror design consisting of flexural springs with a reflecting plate in the center; (**b**) schematic of the electrothermal actuator with bottom Si layer, top Al layer, and embedded Pt heater. The presence of the rectangular notch at the heater end allows for achieving higher vertical deflection in the actuator.

The proposed micromirror design consists of a mirror plate and four bimorph electrothermal actuators, which are symmetrically connected to the mirror plate on four sides through flexural connectors, as shown in Figure 1. The bimorphs consist of two structural layers of aluminum and silicon with an embedded platinum heater. An oxide layer is used for electrical insulation between the structural layers and the heater. The heater pads are exposed to apply the input voltage. To achieve a high out-of-plane displacement, the elecrothermal actuators are optimized with a rectangular notch at the end. Once a voltage difference is applied to the exposed heater pads of a bimorph, a current passes through it and heats it up due to joule heating. The bimorph tends to deflect out-of-plane because of the significant difference in values of CTEs of both constituent structural layers. As a result, the micromirror rotates with a certain angular deflection. For piston mode motion, all four actuators are excited to make a vertical out-of-plane displacement of the micromirror. The micromirror design presented in this paper is optimized considering the microfabrication process presented in [26].

**Figure 2:**
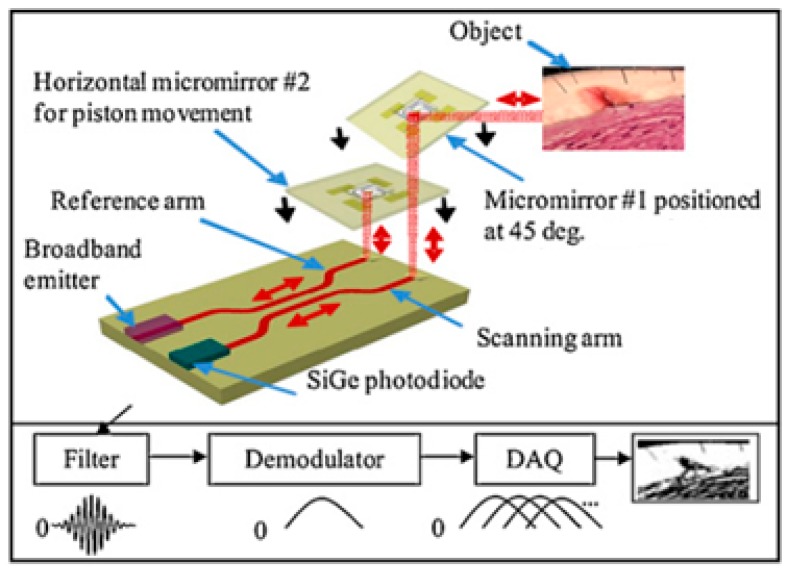
A basic layout of an Optical Coherence Tomography (OCT) system with axial and transverse scanning micromirrors [26].

Figure 2 shows a basic layout of a time domain OCT system consisting of a monolithically integrated broadband light source, silicon germanium photodiode (used as a photodector), waveguides, and micromirrors in the reference and sample arms. In its simplest form, an OCT system operates by splitting a single beam of light into two with a beam splitter. One of the beams travels towards the target sample through the sample arm and the other beam travels to the reference mirror through the reference arm, and reflects back towards the beam splitter from a movable reference mirror. This reflected light from a reference mirror then interacts with the light reflected from the target and produces interference fringes. These signals are then read and electronically processed to determine the reflectivity values of the target as a function of the depth into the tissue. The scanning depth can be swept by changing the path length of the reference mirror.

## 3. Micromirror Design Optimization Using Design of Experiments (DOE)

**Figure 3:**
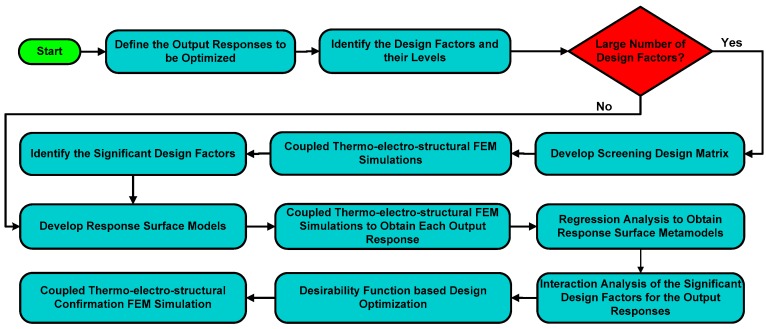
Flowchart showing the schematic layout of the steps implemented for the optimization of the micromirror using Response Surface Models (RSM)-based Design of Experiments (DOE).

A DOE-based design matrix consists of a sequence of FEM simulations to be carried out in terms of design factors set at pre-defined levels. The rows and columns of the design matrix represent the simulation runs and initial design factors settings, respectively. Initially, the identification of the important design factors, affecting a particular output response, is carried out using a screening design matrix. The screening of the significant design factors allows for analyzing and optimizing an output response with respect to design factors in detail using response surface methodology. Figure 3 shows a complete layout for the multi-response optimization using DOE based FEM simulations. The output responses considered in the optimization of the micromirror design, presented in this paper, are the optical deflection angle (twice the mechanical deflection angle), input power, and the temperature rise in micromirror plate from the ambient. Initially, nine design factors that may affect these output responses are considered on two levels, as shown in Table 1. The levels of the design factors are decided based on the previous designs presented in the literature for electrothermally actuated micromirrors. The size of the micromirror plate is considered to be 500 μm × 500 μm, large enough to allow the easy focus of the laser beam spot in optical imaging applications. The low and high levels of the electrothermal actuator length (L) and width (W), shown in Table 1, depict a minimum and maximum L/W ratio of 10 and 16, respectively. The out-of-plane deflection of the actuator can be increased by further increasing this L/W ratio. However, the maximum length of the actuator is limited by the chip size and fill factor, while the width of actuator is dependent on the width of the embedded Pt heater. Moreover, since the thermal response time of the electrothermal actuator is proportional to the square of the actuator length [33], a larger value of actuator length results in lower switching rates for electrothermal micromirrors.

### 3.1. Screening Design Matrix for Significant Design Factors

Screening designs are the most important DOE design matrices that determine the most significant design factors in the optimization process. The Placket–Burman design matrix is the most common screening design matrix used to identify the significant factors in a minimal number of simulation runs with a good degree of accuracy [34]. The Placket–Burman design matrix is based on the first-order model given by:

(1) $$Y=\beta_{0}+\sum_{i=1}^{i=n}\beta_{i}X_{i},$$

where *Y* is the output response, $\beta_{0}$ is the model intercept, $\beta_{i}$ is the linear coefficient, and $X_{i}$ is the level of the design factor. The Placket–Burman design matrix, with 20 simulations and the corresponding three output responses, is shown in Supplementary Table S1. The output responses, for the different combinations of the design factors, are obtained using FEM-based structural-thermal-electric coupled analysis in ANSYS. The structural parts are modeled using SOLID98, which is a coupled field tetrahedral solid element. The micromirror is constrained at the electrothermal actuator ends for both structural and thermal boundary conditions. The material properties used in the FEM simulations are summarized in Table 2. The variation in the material properties with a change in the temperature was previously discussed by the authors in [26] and the temperature coefficient of resistance for the embedded Pt heater in the bimorph actuator was observed to be significantly affected by the temperature. However, in the present work, it is assumed that all the material properties exhibit a linear elastic behavior and remain constant despite changes in the temperature. In general, the heat transfer modes for the electrothermal actuators include conduction, natural convection, and radiation. For a similar micromirror design, presented in [26], the heat transfer due to conduction, convection, and radiation was simulated to be 85%, 14%, and 1%, respectively. These results show a negligible effect of convection and radiation as compared to conduction, and a similar effect has also been presented for electrothermal actuators in [22,35,36,37,38]. Therefore, to reduce computational time during the FEM simulations, only heat transfer due to the conduction is considered, with the assumption that most of the heat transfer occurs along the bimorph actuator and connecting springs as compared to the heat loss from the air. A fixed input voltage of 0.8 V is applied across the electrothermal actuator pads and the corresponding deflection angle, input power, and temperature rise from the ambient in the micromirror plate are obtained. Based on the desired performance of the micromirror, an overall figure of merit (FOM) is obtained considering all three output responses:

(2) $$\mathrm{FOM}=\frac{\text{Deflection angle}}{\text{Input power}\;\left(\mathrm{mW}\right)\times \text{Temperature rise from the ambient}\;\left({}^{\circ}C\right)}.$$

### 3.2. Mean Effect Model and Analysis of Variance for the Screening Design

**Figure 4:**
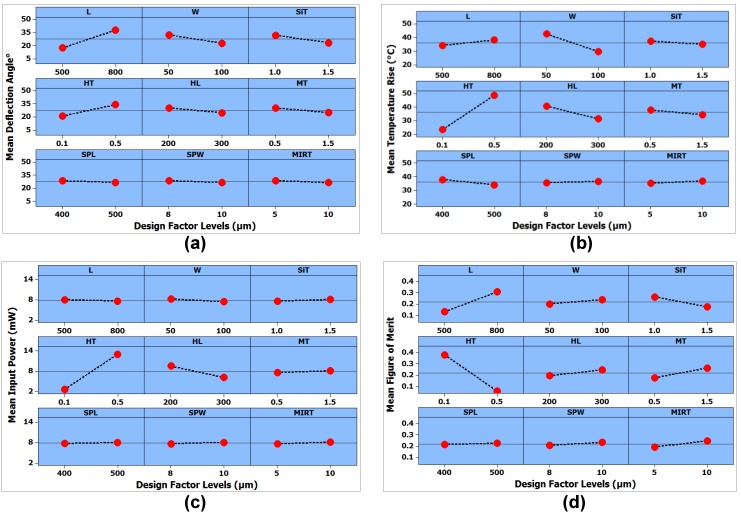
Mean effect plots for the output responses: (**a**) mean effect plot for the deflection angle. Design factor actuator length (L) has the highest change in the mean value with change from low level to high level; (**b**) Mean effect plot for the micromirror central plate temperature rise from the ambient. The design factor heater thickness (HT) has a highest deviation from the mean; (**c**) Mean effect plot of the input power. The design factor HT and heater length (HL) have a visible change in the mean values while all other factors have negligible effect on mean at two different levels; (**d**) Mean effect plot of the figure of merit showing the design factors HT and L to have the highest change in the mean value going from the low to high factor level.

The output responses obtained using the FEM simulations for the screening design matrix can be described by a linear statistical model given as:

(3) $$y_{ij}=\mu_{i}+\varepsilon_{ij}\{\begin{array}{c} i=1,2,,,a\\ j=1,2,,,n \end{array},$$

where $y_{ij}$ is the *ijt*h response value, *n* is the number of times the design factor level appears in the design matrix, $\mu_{i}$ is the *ijt*h design factor level, and $\varepsilon_{ij}$ are the random errors. The means for each design factor at low and high level are obtained for each output response, as shown in Figure 4. The horizontal axis for each design factor is the low and high level value, while the vertical axis is the mean value of the output responses for each design factor level. A large difference in the means of two design factor levels shows that the design factor has a significant effect on the output response. Figure 4a shows a steeper slope for the two levels of the design factors L, W, SiT, MT, HL, and HT. Figure 4b shows that the only heater length (HL), heater thickness (HT), and actuator width (W) have a significant effect on the temperature rise of the micromirror plate. For the input power, the slope of the heater length (HL) and heater thickness (HT) is high compared to the other design factors, as shown in Figure 4c. Since the mean effect of the design factors at low and high levels is not the same for all three output responses, the mean effect plot for the figure of merit is obtained using Equation (2). Figure 4d shows the mean effect plots of the design factors for the overall FOM.

**Figure 5:**
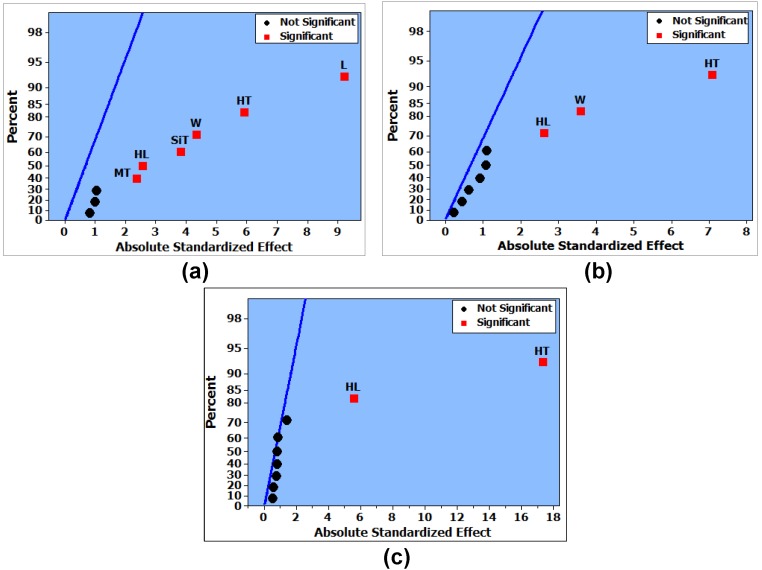
(**a**) Half-normal probability plot for deflection angle. The effect of the actuator length (L) is highest among the significant factors, while that of the mirror thickness (MT) is lowest; (**b**) Half-normal probability plot for the micromirror temperature rise from the ambient. Among the three significant factors, heater thickness (HT) has the highest effect on temperature rise and silicon thickness (SiT) has the lowest; (**c**) Half-normal probability plot of the input power. Heater thickness (HT) and heater length (HL) are the two significant design factors.

Analysis of variance (ANOVA) is a collection of statistical design models that analyze the effect of considered design factors on a specific output response. This technique is based on the assumption that the sources of variability in the output response variables can be attributed to the design factors as well as to the random noise in the experiments. The total variation in the output response for each design factor is calculated in the form of the total sources of variance *SS_T_*, which is a combination of variable sum of squares (variance due to design factors effects) *SS_A_* and error sum of squares (random error) *SS_E_*. These total source variance can be represented as [41]:

(4) $$SS_{T}=SS_{A}+SS_{E}$$

(5) $$SS_{A}=\overset{a}{\underset{i=1}{\sum }}\overset{n_{i}}{\underset{j=1}{\sum }}{\left({\bar{y}}_{i}-\bar{y}\right)}^{2}$$

(6) $$SS_{E}=\sum_{i=1}^{a}\sum_{j=1}^{n_{i}}{\left(y_{ij}-{\bar{y}}_{i}\right)}^{2},$$

where ${\bar{y}}_{i}$ is the design factor level group mean, $\bar{y}$ is the overall mean, a is the number of levels of the design factor, $y_{ij}$ is the the *ijt*h response in the *it*h variable level, and $n_{i}$ is the number for which the variable is at *i* level. The assumptions for ANOVA (that the random errors are normally distributed with mean zero and constant variance) are initially verified using Anderson–Darling [42] and Levene tests [41]. A detailed description of these tests is provided by the authors in [43]. *p*-values ˃ 0.05 are obtained for both these tests, thus verifying the basic ANOVA assumptions. ANOVA results are generally described in terms of *p*-value. A *p*-value ≤ 0.05 for a design factor means that it can be concluded with 95% confidence level that the considered design factor has a significant effect on the output response. For the angular deflection, *p*-value ≤ 0.05 is obtained for the design factors L, W, SiT, HT, HL, and MT. Similarly, the analysis showed *p*-value ≤ 0.05 for the design factors W, HT, and HL in the case of micromirror temperature rise from ambient. For the input power, the design factors HT and HL showed *p*-value ≤ 0.05. The results obtained using ANOVA are further verified using half-normal probability plots [44]. The half-normal probability plots are used to find out whether and to what extent the distribution of the design factors follow the normal distribution. The estimates for the significant design factors do not follow the normal distribution. Figure 5 shows the half-normal probability plots for the three output responses of the screening design matrix.

### 3.3. Design Matrix for Multi-Response Optimization

The ANOVA and half-normal probability plots show that the significant design factors for the angular deflection also include the design factors that were proven to be significant for the micromirror temperature rise and input power. So, the significant design factors L, W, HL, HT, SiT, and MT, obtained using a Plackett–Burman based screening design matrix for the angular deflection, are further investigated using response surface metamodels. The response surface method is based on a statistical approach to develop an appropriate relationship between an output response and the design factors using the following second-order model:

(7) $$\begin{array}{ccc} y=\beta_{0}+\sum_{i=1}^{k}\beta_{i}x_{i}+\sum \sum_{i<j}\beta_{ij}x_{i}x_{j}+\sum_{i=1}^{k}\beta_{ii}x_{i}^{2}+\epsilon & & \left(d=2\right), \end{array}$$

where ϵ is the random error and the β coefficients are obtained by the method of least squares regression, such that the sum of the squares of the predicted values and the actual values are minimized. In matrix form, Equation (7) can be written as:

(8) Y=bX+E,

where ***Y*** is the matrix of the measured output response values and ***X*** is the matrix of the design factors. The matrix ***b*** of the β coefficients can be obtained as:

(9) $$b={\left(X^{T}X\right)}^{-1}X^{T}Y.$$

The selection of a proper design matrix for the response surface-based optimization is very important. In this work, we have selected Central Composite Design (CCD) design matrix for multi-response optimization. The CCD requires only a fraction of all the possible combinations of the design factors. The number of simulation runs required for the CCD design matrix are *N* = *2^k^* + *2k* + *C*_0_ where *k* is the number of the design factors and *C*_0_ is the number of central points. Table 3 shows the significant design factors at three levels used for the response surface metamodels.

The CCD design matrix with 53 simulation runs and the corresponding output responses (deflection angle, input power, and temperature rise in the mirror) obtained through FEM simulations are shown in Supplementary Table S2. The non-significant design factors SpL, SpW, and MIRT are kept at the levels that gave a maximum value of FOM in the Plackett–Burman screening design. Polynomial equations for the responses *Y*_1_ = deflection angle, *Y*_2_ = Input power, *Y*_3_ = micromirror temperature rise obtained, using the second-order model and calculating the value of the β coefficients (using Equations (7)–(9)), are given as:

(10) $$\begin{array}{l} Y_{1}=23.59+9.86X_{1}+7.39X_{2}-4.66X_{3}-5.23X_{4}-4.62X_{5}-1.02X_{6}+2.84X_{1}X_{2}-\\ 1.24X_{1}X_{3}-1.81X_{1}X_{4}-1.27X_{1}X_{5}+0.048X_{1}X_{6}-1.59X_{2}X_{3}-1.03X_{2}X_{4}-\\ 0.88X_{2}X_{5}-0.24X_{2}X_{6}+0.97X_{3}X_{4}+0.67X_{3}X_{5}+0.29X_{3}X_{6}+0.89X_{4}X_{5}+2.10X_{4}X_{5}+\\ 0.088X_{5}X_{6}+0.37X_{1}^{2}-1.52X_{2}^{2}+5.71X_{3}^{2}+0.28X_{4}^{2}+0.60X_{5}^{2}-1.94X_{6}^{2}. \end{array}$$

(11) $$\begin{array}{c} Y_{2}=7.48+5.18X_{2}-0.20X_{3}-1.65X_{5}-0.13X_{2}X_{3}-1.10X_{2}X_{5}+0.080X_{3}X_{5}\\ -2.953e^{-4}X_{1}^{2}+2.047e^{-4}X_{2}^{2}-0.075X_{3}^{2}-2.953e^{-4}X_{4}^{2}+0.38X_{5}^{2}-2.953e^{-4}X_{6}^{2} \end{array}$$

(12) $$\begin{array}{c} Y_{3}=27.06-0.17X_{2}+13.65X_{2}-7.50X_{3}-1.33X_{4}-5.64X_{5}-3.70X_{6}+0.1X_{1}X_{2}-\\ 0.015X_{1}X_{3}+9.906e-003X_{1}X_{4}+0.026X_{1}X_{5}+0.16X_{1}X_{6}-3.35X_{2}X_{3}-0.70X_{2}X_{4}-\\ 1.64X_{2}X_{5}-2.15X_{2}X_{6}+0.53X_{3}X_{4}+1.21X_{3}X_{5}+1.32X_{3}X_{6}+0.24X_{4}X_{5}+0.77X_{4}X_{6}+\\ 0.38X_{5}X_{6}-0.33X_{1}^{2}-0.030X_{2}^{2}+7.68X_{3}^{2}-0.23X_{4}^{2}+0.62X_{5}^{2}+0.74X_{6}^{2}. \end{array}$$

### 3.4. Regression Analysis for the CCD Design Matrix

To verify that the developed response surface models for the three output responses, given in Equations (10)–(12), provide an adequate approximation of the true behavior of the micromirror, a regression analysis is carried out. The first step in the regression analysis is to ensure that none of the least squares assumptions, i.e., errors in the model, are normally distributed with mean zero and variance $\sigma^{2}$, are violated [41]. These assumptions are verified by analyzing the residuals from the least squares fit defined by $e_{i}=y_{i}-{\hat{y}}_{i}$, *i* = 1, 2, ..., *n,* where $y_{i}$ is the vector of actual observed values and ${\hat{y}}_{i}$ is the vector of the fitted values. The relationship of the vector of fitted values to the vector of actual observed values is given as:

(13) $$\hat{y}=Xb=X{\left(X^{T}X\right)}^{-1}X^{T}y.$$

To verify the assumptions for the response surface models obtained from the CCD design matrix, standardized residuals-based normal probability plots and fitted values versus standardized residual plots are obtained, which verified the basic assumptions for the regression analysis. The test for the regression analysis is based on the following null hypothesis [41]:

(14) $$\begin{array}{r} H_{0}:\beta_{1}=\beta_{2}=\ldots =\beta_{k}=0\\ H_{1}:\beta_{j}\neq 0\;\text{for at least one}\;j \end{array}\}.$$

The regression analysis test is used to verify if a statistical relationship exists between the output response and at least one of the design factors. If the null hypothesis is rejected, then it means that at least one of the design factors significantly affects the output response surface model. The test for the hypothesis is carried out using the following F-test ratio:

(15) $$F_{0}=\frac{MS_{R}}{MS_{E}},$$

where $MS_{R}$ and $MS_{E}$ are the regression and residual mean square, respectively. The null hypothesis is rejected if the calculated $F_{0}>F_{\alpha ,k,n-k-1}$ (or *p*-value < α), where α is the level of significance, *k* is the number of design factors, and *n* is the number of observations [41]. A regression analysis for the three output responses is performed and *F*-test ratios and corresponding *p*-values for each output response model are obtained. Supplementary Table S3 shows the regression analysis results.

### 3.5. Interaction Analysis of the Design Factors for Angular Deflection

**Figure 6:**
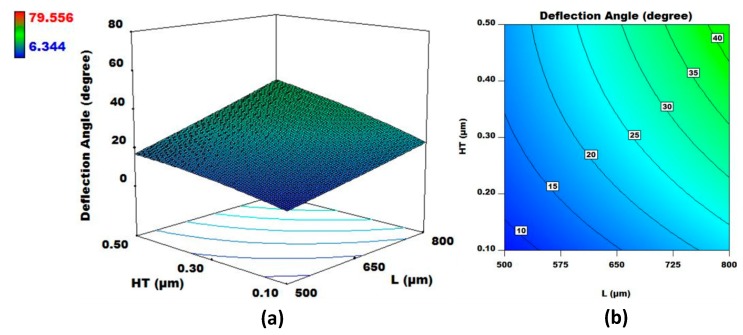
(**a**) 3D surface plot and (**b**) contour plot for L and HT for fixed values of W, HL, SiT, and MT. The output response considered is angular deflection.

The regression analysis results, given in Supplementary Table S3, for the output response angular deflection ($Y_{1}$) show that the design factor interactions $X_{1}X_{2}$, $X_{1}X_{3}$, $X_{1}X_{4}$, $X_{1}X_{5}$, $X_{2}X_{3}$, $X_{2}X_{4}$, $X_{2}X_{5}$, $X_{3}X_{4}$, $X_{3}X_{5}$, $X_{4}X_{5}$, and $X_{4}X_{6}$ are significant with *p*-value < 0.05. These interactions for the design factors can be further analyzed with respect to the output response using 3D surface and contour plots. In this paper, the design factor interaction $X_{1}X_{2}$ with the highest *F*-value of 83.2 is further investigated using 3D surface and contour plots as an example. Figure 6 shows that the deflection angle increases with an increase in both the electrothermal actuator length L ($X_{1}$) and heater thickness HT ($X_{2}$). The deflection angle is more sensitive to the change in L as compared to HT. When the actuator length is 500 μm, the change in the deflection angle is less affected by the change in the heater thickness as compared to when the actuator length is at 800 μm.

### 3.6. Interaction Analysis of the Design Factors for Input Power

**Figure 7:**
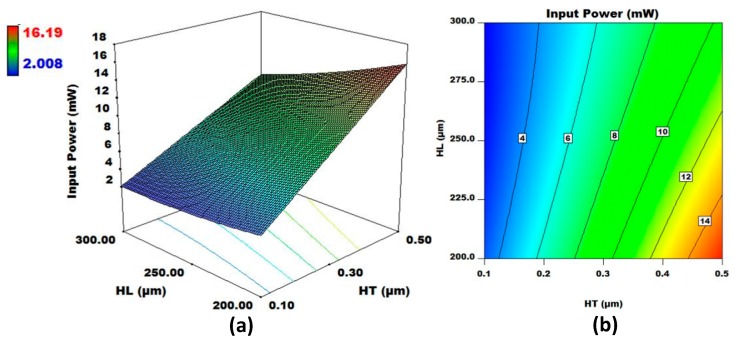
(**a**) 3D surface plot and (**b**) contour plot for HL and HT for fixed values of W, L, SiT, and MT. The output response considered is input power.

In Supplementary Table S3, the *p*-values < 0.05 for $X_{2}X_{3}$, $X_{2}X_{5}$, and $X_{3}X_{5}$ show that there is a significant relationship between the W and HT, HT and HL, and HL and W for the output response input power ($Y_{2}$). The interaction between HT and HL has the highest *F*-value among the three significant design factor interactions. Figure 7 shows the 3D surface and contour plots for HT and HL, with all other design factors set at their medium levels. The plots show that input power decreases with the increase in the heater length and a decrease in the heater thickness. The change in the output power is more sensitive to the change in the heater thickness as compared to the heater length. Moreover, the change in the input power value is less than the change in the HL when HT = 0.1 μm, as compared to when HT = 0.5 μm.

### 3.7. Interaction Analysis of the Design Factors for Temperature Rise

**Figure 8:**
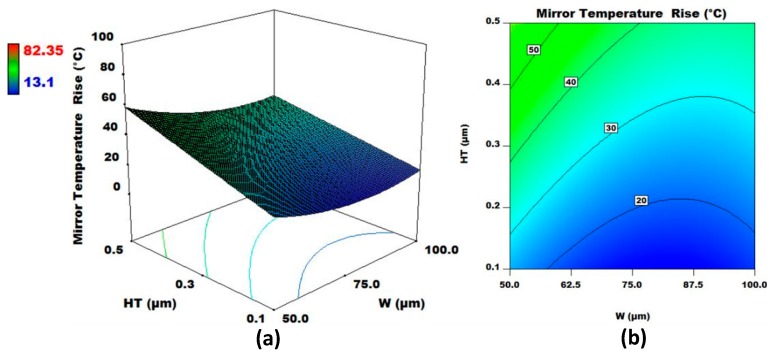
(**a**) 3D surface plot and (**b**) contour plot for W and HT for fixed values of L, HL, SiT, and MT. The output response considered is micromirror central plate temperature rise from the ambient.

The design factor interactions $X_{2}X_{3}$, $X_{2}X_{5}$, $X_{2}X_{6}$, $X_{3}X_{5}$, $X_{3}X_{6}$, and $X_{4}X_{6}$ are observed to be the significant interactions for the temperature rise in the micromirror plate from the ambient. Figure 8 shows the 3D surface and contour plots for the interaction between the electrothermal actuator width W ($X_{3}$) and heater thickness HT ($X_{2}$). The interaction between W and HT has the highest *F*-value of 117.4 for the temperature rise as compared to all other significant design factor interactions. The interaction plots between W and HT are highly non-linear, with a noticeable curvature. The plots show that the temperature rise in the micromirror plate is less sensitive to the change in the electrothermal actuator width W as compared to the heater thickness HT for a fixed value of all other design factors. However, for W ≤ 75 μm, the temperature rise in the micromirror is more influenced by the change in the HT as compared to when 75 μm ≤ W ≤ 100 μm.

### 3.8. Multi-Response Optimization

The design optimization of electrothermally actuated micromirror, considered in this paper, involves optimization of three output responses simultaneously for a given set of design factors. For the multi-response optimization of the micromirror, an optimization objective function is initially defined, which is given as:

(16) $$\begin{array}{c} \text{ Maximize deflection angle}\\ \text{Minimize input power}:\\ \text{Temperature rise in the micromirror plate}\leq 30\;{}^{\circ}C\\ \text{such that}:\\ 500\;\mu m\leq L\leq 800\;\mu m\\ 0.1\;\mu m\leq \mathrm{HT}\leq 0.5\;\mu m\\ 50\;\mu m\leq W\leq 100\;\mu m\\ 1\;\mu m\leq \mathrm{SiT}\leq 1.5\;\mu m\\ 200\;\mu m\leq \mathrm{HL}\leq 300\;\mu m\\ 0.5\;\mu m\leq \mathrm{MT}\leq 1.5\;\mu m. \end{array}$$

One of the traditional methods for multi-response optimization is overlaid contour plots. This method is mainly useful when there are two or three design factors, since in higher dimensions it loses its efficiency [45]. The most practical method to optimize multiple output responses was proposed by Derringer and Suich and is based on the desirability function approach [46]. The desirability function allows us to find suitable values for the design factors to simultaneously reach an optimal solution for all the output responses considering the desired objective function. Initially, an individual desirability function $d_{i}\left(y_{i}\right)$ for each response $y_{i}$ is calculated using the developed response surface models and defined objective function. If the output response $y_{i}$ is at the goal defined in the objective function then $d_{i}$ = 1, and if it is outside an acceptable region then $d_{i}$ = 0. If the objective function is to maximize the output response then $d_{i}\left(y_{i}\right)$ is given as [45]:

(17) $$d_{i}\left(y_{i}\left(x\right)\right)=\{\begin{array}{cccc} 0 & & & \mathrm{if}\;y_{i}\left(x\right)<L_{i}\\ {\left(\frac{y_{i}\left(x\right)-L_{i}}{U_{i}-L_{i}}\right)}^{r_{1}} & & & \mathrm{if}\;L_{i}\leq y_{i}\left(x\right)\leq U_{i}\\ 1 & & & \mathrm{if}\;y_{i}\left(x\right)>U_{i} \end{array}.$$

If the objective function is to minimize the output response then $d_{i}\left(y_{i}\right)$ is given as:

(18) $$d_{i}\left(y_{i}\left(x\right)\right)=\{\begin{array}{cccc} 1 & & & \mathrm{if}\;y_{i}\left(x\right)<L_{i}\\ {\left(\frac{U_{i}-y_{i}\left(x\right)}{U_{i}-L_{i}}\right)}^{r_{2}} & & & \mathrm{if}\;L_{i}\leq y_{i}\left(x\right)\leq U_{i}\\ 0 & & & \mathrm{if}\;y_{i}\left(x\right)>U_{i} \end{array}.$$

When the output response is to be optimized with respect to some target *T* then $d_{i}\left(y_{i}\right)$ is given as:

(19) $$d_{i}\left(y_{i}\left(x\right)\right)=\{\begin{array}{cccc} 0 & & & \mathrm{if}\;y_{i}\left(x\right)<L_{i}\\ {\left(\frac{y_{i}\left(x\right)-L_{i}}{T_{i}-L_{i}}\right)}^{r_{1}} & & & \mathrm{if}\;L_{i}\leq y_{i}\left(x\right)\leq T_{i}\\ 1 & & & \mathrm{if}\;y_{i}\left(x\right)=T_{i}\\ {\left(\frac{y_{i}\left(x\right)-U_{i}}{T_{i}-U_{i}}\right)}^{r_{2}} & & & \mathrm{if}\;T_{i}\leq y_{i}\left(x\right)\leq U_{i}\\ 0 & & & \mathrm{if}\;y_{i}\left(x\right)>U_{i} \end{array},$$

where $U_{i}$ is the upper value of the desired output response range, $L_{i}$ is the lower value of the output response range, and $T_{i}$ is the target value for the output response. The parameters $r_{1}$ and $r_{2}$ define the importance of the output response to be close to the desired value. The optimum solution can be obtained by combining the individual desirability functions, given as:

(20) $$D\left(d_{1}\left[y_{1}\left(x\right)\right],d_{2}\left[y_{2}\left(x\right)\right],\cdots ,d_{n}\left[y_{n}\left(x\right)\right]\right)={\left(\prod_{i=1}^{n}d_{i}\left[y_{n}\left(x\right)\right]\right)}^{\frac{1}{n}}.$$

**Figure 9:**
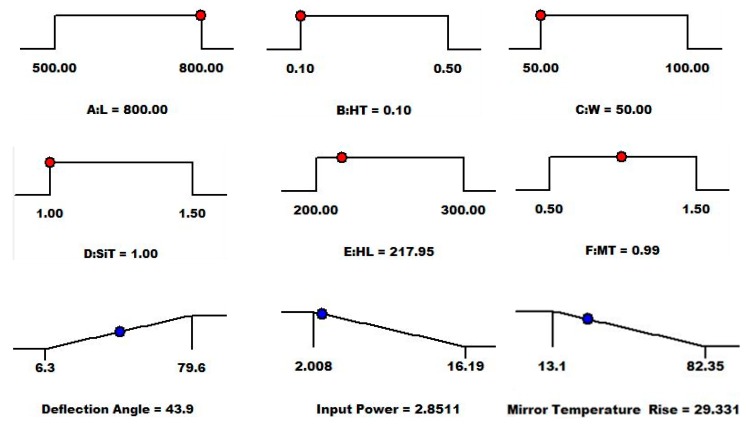
The optimal values of the design factors obtained using the Nelder–Mead downhill simplex-based heuristic search algorithm.

The desirability values for the multiple output responses can be maximized by using the well-known Nelder–Mead downhill simplex algorithm-based heuristic search algorithm [47]. This search algorithm finds a local optimum solution to a problem with multiple variables and iteratively narrows down to a design factor value that maximizes the desirability of the objective function. Figure 9 shows the optimal solution for three output responses and the corresponding values of the design factors with respect to the optimization objective function defined in Equation (14). The values of the simultaneously optimized deflection angle, input power, and micromirror temperature rise from the ambient are 43.9°, 2.85 mW, and 29.3 °C, respectively. The value of the combined overall desirability function is 0.72. 

**Figure 10:**
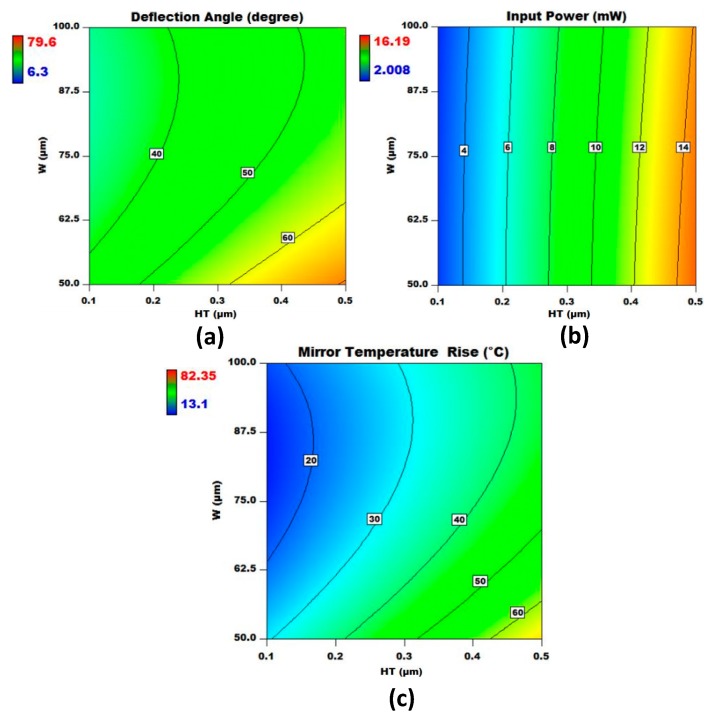
Contour plots for (**a**) deflection angle; (**b**) input power; and (**c**) micromirror plate temperature rise from the ambient. The contour plots show interaction between W and HT for the final optimized design, while all other design factors are kept at the optimized values predicted by the direct search algorithm.

The regression analysis results for all three output responses, given in Supplementary Table S3, show that the interaction between the design factors W and HT is a significant interaction with *p*-value < 0.05. This gives an opportunity to further explore the effect of these two factors on the individual output responses and overall desirability by keeping all other design factors at the optimized values predicted by the desirability function approach. Figure 10 shows the contour plots for the effect of W and HT on the deflection angle, input power, and micromirror temperature rise from the ambient. For the deflection angle, the interaction between the electrothermal actuator width W and heater thickness HT is highly non-linear, with large contours. The deflection angle increases considerably with the increase in the HT up to 0.5 μm. The change in the deflection angle is more sensitive to the change in HT than W. For the input power, the contour plot shows linear behavior. The input power changes sharply with the increase in the HT, while the effect of the change in W is negligible. The contour plots for the micromirror temperature rise from the ambient show that the output response is very sensitive to the increase in HT as compared to W. However, for W ≥ 62.5 μm, change in W has an almost negligible effect on the temperature rise.

**Figure 11:**
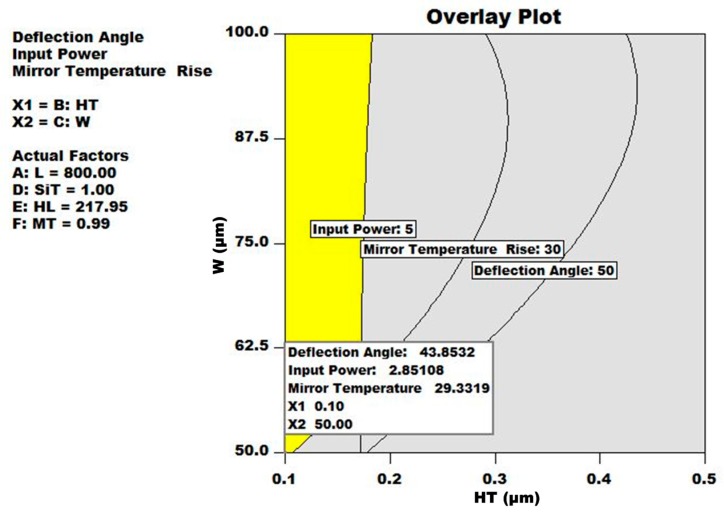
Overlay contour plots for all three output responses. The yellow region shows the acceptable solutions according to the objective function defined in Equation (16). However, the best solution with highest desirability is highlighted in the text box.

Figure 11 shows the overlay contour plots for the three output responses with respect to HT and W. The individual contour plots are obtained using response surface models by using the predicted design factor values. The yellow region shows all the feasible solutions that lie within the defined objective function of Equation (16).

### 3.9. Verification of the Multi-Response Optimization

**Figure 12:**
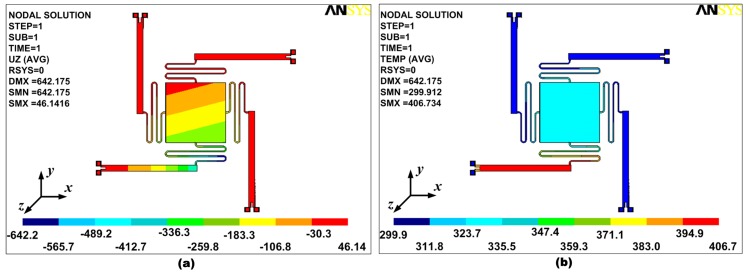
(**a**) Vertical deflection plot for the final optimized design; (**b**) temperature distribution within the final optimized design. The temperature rise in the micromirror plate is much less than the bimorph actuator temperature.

The results obtained using desirability function-based multi-response optimization are verified using FEM simulations. The micromirror model is developed with the optimal values of the design factors, shown in Figure 9. Figure 12a shows the vertical deflection in the micromirror plate with a maximum upward and downward deflection of 18.4 μm and 263.4 μm respectively, in opposite corners of the micromirror plate. An absolute deflection angle of 43.4° is calculated for the micromirror plate deflection using trigonometric functions. Figure 12b shows the temperature distribution in the micromirror. The temperature rise in the micromirror plate from the ambient is 27.4 °C at an actuation voltage of 0.8 V. The calculated input power for the optimized design for an actuation voltage of 0.8 V is 2.94 mW. These actual values of the deflection angle, micromirror plate temperature rise from ambient, and input power, obtained using FEM simulations, lie within the 95% confidence interval of the predicted output responses, thus verifying the accuracy of the developed response surface models-based multi-response optimization.

**Figure 13:**
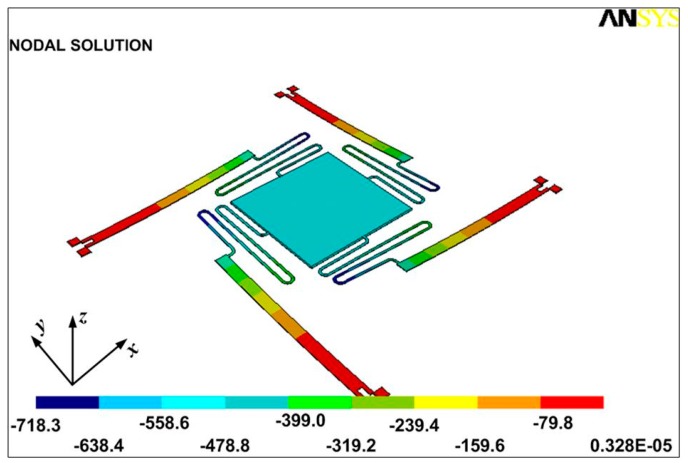
Vertical deflection in the final optimized design for piston mode operation. All four electrothermal actuators are simultaneously actuated.

The DOE-based optimization technique discussed in this paper is implemented only for the scanning micromirror used in the sample arm of an OCT system. However, for the use of a micromirror in the reference arm of an OCT, or for in-depth tissue scanning, an out-of-plane displacement (piston mode operation) is desired. To analyze the possibility of using the optimized tilting micromirror in the reference arm of an OCT system, all four bimorph actuators are simultaneously actuated in the FEM simulations. A vertical displacement of nearly 500 μm is obtained in the micromirror plate, as shown in Figure 13. The temperature rise in the micromirror plate from the ambient is observed to be 110 °C for the piston mode, which is much higher than the case of angular deflection.

## 4. Discussion

The output responses considered in the optimization of the electrothermal micromirror using Response Surface Models (RSM) are deflection angle, input power, and micromirror temperature rise from the ambient. However, for an OCT system, the scanning speed and overall device area of the electrothermal micromirror are also important output responses. The scanning speed is dependent on the thermal time response of the bimorph actuator, while the overall device area is decided by the electrothermal actuator and micromirror reflecting plate dimensions. These output responses may also be considered in the multi-response optimization of the electrothermal micromirror, following the optimization steps discussed earlier. For the final optimized design presented in this paper, the overall micromirror size is 1.65 mm × 1.65 mm, with a mirror plate size of 500 μm × 500 μm. For a fixed mirror plate size, the device area may be minimized by decreasing the electrothermal actuator length. For example, with an actuator length of 400 μm, the overall device area is 0.825 mm × 0.825 mm and the corresponding RSM-based deflection angle, input power, and micromirror temperature rise from the ambient are 18.3°, 3.3 mW and 34.8 °C, respectively. These values are obtained by modifying the objective function given in Equation (16) with L = 400 μm and repeating all the optimization steps.

The performance of metal thin-films-based MEMS devices is significantly affected by the time-dependent accumulation of plastic strain under the influence of applied stress and temperature (the creep effect). This leads to a change in both the static and dynamic response of the device [48]. In MEMS, the creep effect was initially reported in electrostatically actuated digital micromirror devices (DMD), fabricated using a series of aluminum metal depositions [49]. The micromirrors were tested at a temperature of 65 °C and a change in the static response was observed. In the electrothermally actuated micromirrors, the temperature in the actuators is generally higher than room temperature. Mu et al. [50] have reported a temperature of 90 °C in a Al/Si bimorph thermal actuator with a relatively low temperature of 30 °C in the mirror plate. Bauer et al. [51] have implemented Au/Si bimorph actuators in a scanning micromirror design for an initial offset of the angular vertical comb-drives. The temperature in the bimorph actuators is simulated to be 380 °C, while a very high temperature of 770 °C in the comb-drive supporting beam is reported. For an electrothermally actuated micromirror, temperature values of nearly 160 °C and 65 °C in the Al/W bimorph actuator and mirror plate, respectively, are reported in [14]. For the optimized micromirror design, presented in this paper, the temperature distribution in the micromirror (Figure 12) shows a temperature increase of nearly 106 °C and 27.4 °C from the ambient in the bimorph actuator and micromirror plate, respectively. These high temperature values in the metal thin-film-based electrothermal micromirrors may initiate the creep phenomenon and affect long-term reliability. The other mechanical reliability issue related to micromirrors is the formation of residual stress during the microfabrication process, which may result in curling in both the thermal actuators and central plate, like all other MEMS devices [52]. Similarly, the application areas of the micromirrors involve cyclic loading, which may deteriorate the flexural stiffness if the device is operated for a large number of cycles (the fatigue phenomenon) [53]. Generally, during the design and optimization phase of the MEMS in general, and electrothermal micromirrors in particular, these reliability issues are not considered. A reliable design of an electrothermally actuated micromirror requires a robust design optimization considering both thermal and mechanical reliability issues. A DOE-based robust multi-response-based design optimization using the dual response surface method [54] or mixed array design [36] can be a good alternative to the conventional design optimization methodologies for electrothermal micromirrors.

## 5. Conclusions

A DOE-based multi-response design optimization methodology for MEMS devices is presented. The device considered for optimization is an electrothermally actuated micromirror for OCT system applications. Three output responses, deflection angle, input power, and micromirror temperature rise from the ambient, are considered for simultaneous optimization and the respective response surface models are developed through regression analysis. A desired objective function is defined and the optimal values of the design factors and corresponding output responses, satisfying the objective function, are obtained using combined desirability functions and a Nelder–Mead downhill simplex-based heuristic search algorithm. A deflection angle of 44° with an input power of 2.85 mW and a temperature rise of 29.3 °C from ambient, with an overall device size of 1.65 mm × 1.65 mm, is predicted by the developed RSM model at the optimal level of the design factors. These predicted values are verified using FEM-based confirmation simulation. The proposed multi-response design optimization methodology can be implemented for the optimization and detailed interaction analysis of different design factors of MEMS devices at the design level.

## Figures and Tables

**Table 1:** Design factors with their respective codes, selected at two levels, for the optimization of the micromirror.

Code	Design Factor (μm)	Low Level (−1)	High Level (+1)
X_1_	Actuator Length (L)	500	800
X_2_	Actuator Width (W)	50	100
X_3_	Silicon Thickness (SiT)	1	1.5
X_4_	Heater Thickness (HT)	0.1	0.5
X_5_	Heater Length (HL)	200	300
X_6_	Metal Thickness (MT)	0.5	1.5
X_7_	Spring Length (SpL)	400	500
X_8_	Spring Width (SpW)	8	10
X_9_	Mirror Thickness (MIRT)	5	10

**Table 2:** Material properties used in the FEM simulations [22,24,39,40].

Material Properties	Aluminum	Platinum	Silicon	Silicon Dioxide
Young’s modulus (GPa)	70	170	162	70
Poisson ratio	0.33	0.38	0.22	0.17
Density (kg/µm^3^)	2.3 × 10^−15^	21.4 × 10^−15^	2.32 × 10^−15^	2.66 × 10^−15^
Specific heat (pJ/kg K)	9.02 × 10^14^	1.3 ×1 0^14^	7.53 × 10^14^	10 × 10^14^
Resistivity (TΩ·µm)	2.83 × 10^−14^	10.9 × 10^−14^	1.32 × 10^−14^	1.0 × 10^10^
CTE (1/K)	23.1 × 10^−6^	8.8 × 10^−6^	2.66 × 10^−6^	0.5 × 10^−6^
Thermal conductivity (pW/µm K)	23.7 × 10^7^	7.1 × 10^7^	1.5 × 10^8^	0.1 × 10^7^

**Table 3:** Design factors and their three levels for the Central Composite Design (CCD) design matrix.

Code	Design Factor (um)	Low Level (−1)	Medium Level (0)	High Level (+1)
*X*_1_	Actuator Length (L)	500	650	800
*X*_2_	Heater Thickness (HT)	0.1	0.3	0.5
*X*_3_	Actuator Width (W)	50	75	100
*X*_4_	Silicon Thickness (SiT)	1.0	1.25	1.5
*X*_5_	Heater Length (HL)	200	250	300
*X*_6_	Metal Thickness (MT)	0.5	1.0	1.5

## Supplementary Materials

Supplementary File 1
