# Supplementary material for: Multi-Response Optimization of Electrothermal Micromirror Using Desirability Function-Based Response Surface Methodology
Source: Micromachines (Basel). 2017 Apr 1;8(4):107. doi: 10.3390/mi8040107 (PMC6189808; doi:10.3390/mi8040107)
Supplement: Supplementary File 1 [file micromachines-08-00107-s001.pdf]

micromachines

Supplementary Materials: Multi-Response Optimization of Electrothermal Micromirror Using Desirability Function-Based Response Surface Methodology

Muhammad Mubasher Saleem, Umar Farooq, Umer Izhar, and Umar Shahbaz Khan

Table S1. Screening design matrix with 20 simulation runs and the corresponding output responses obtained using Finite Element Modeling (FEM) simulations.

Run No.	X_1	X_2	X_3	X_4	X_5	X_6	X_7	X_8	X_9	Deflection angle (Degree)	Temperature rise (°C)	Input power (mW)	Figure of Merit
1	+1	−1	−1	−1	−1	+1	−1	+1	−1	40.5	28.2	3.2	0.443
2	+1	+1	+1	+1	−1	−1	+1	+1	−1	35.0	46.0	15.2	0.049
3	−1	−1	−1	−1	+1	−1	+1	−1	+1	15.7	22.8	2.1	0.329
4	+1	−1	+1	+1	−1	−1	−1	−1	+1	51.0	72.6	16.2	0.043
5	−1	−1	+1	+1	−1	+1	+1	−1	−1	25.6	59.6	16.1	0.026
6	+1	+1	−1	+1	+1	−1	−1	−1	−1	45.6	39.4	10.0	0.115
7	+1	−1	+1	−1	+1	+1	+1	+1	−1	21.2	19.3	2.0	0.526
8	−1	−1	−1	−1	−1	−1	−1	−1	−1	25.6	36.2	3.3	0.218
9	+1	+1	−1	−1	−1	−1	+1	−1	+1	36.8	22.3	3.1	0.540
10	−1	+1	−1	+1	+1	+1	+1	−1	−1	14.7	29.5	10.0	0.049
11	−1	+1	+1	+1	+1	−1	−1	+1	+1	11.0	34.9	10.0	0.031
12	−1	+1	−1	+1	−1	+1	+1	+1	+1	22.6	40.8	15.3	0.036
13	−1	+1	+1	−1	+1	+1	−1	−1	−1	6.4	13.1	2.0	0.244
14	−1	+1	+1	−1	−1	−1	−1	+1	−1	9.5	21.4	3.1	0.146
15	+1	−1	+1	+1	+1	+1	−1	−1	+1	40.1	42.6	10.4	0.090
16	+1	−1	−1	+1	+1	−1	+1	+1	−1	61.6	61.0	10.3	0.096
17	−1	−1	+1	−1	+1	−1	+1	+1	+1	8.6	21.5	2.1	0.191
18	+1	+1	+1	−1	−1	+1	+1	−1	+1	23.6	19.4	3.0	0.398
19	−1	−1	−1	+1	−1	+1	−1	+1	+1	32.5	61.9	16.1	0.032
20	+1	+1	−1	−1	+1	+1	−1	+1	+1	20.9	30.6	2.0	0.769

Table S2. Central Composite Design (CCD) design matrix with 53 simulation runs. Output responses are obtained for each simulation run using FEM.

Run	X_1	X_2	X_3	X_4	X_5	X_6	Y_1 Deflection angle (°)	Y_2 Input power (mW)	Y_3 Temperature rise (°C)
1	+1	−1	−1	+1	−1	−1	25.84	3.23	30.65
2	−1	+1	+1	−1	+1	+1	14.77	10.04	29.70
3	−1	−1	−1	−1	−1	−1	25.53	3.23	36.50
4	+1	+1	+1	−1	−1	+1	49.88	15.26	40.93
5	+1	−1	+1	−1	−1	−1	35.54	3.05	21.80
6	−1	−1	−1	−1	+1	+1	12.11	2.08	19.26
7	0	0	0	0	0	0	23.53	7.48	26.98
8	0	0	0	−1	0	0	28.41	7.48	27.93
9	−1	−1	+1	+1	+1	+1	6.34	2.00	13.10
10	+1	−1	−1	−1	−1	+1	39.82	3.23	28.10
11	+1	+1	−1	−1	+1	+1	51.11	10.43	45.50
12	0	0	0	0	0	0	23.53	7.48	26.98
13	+1	−1	+1	+1	−1	+1	23.08	3.05	19.26
14	0	0	0	0	0	0	17.89	7.48	26.98
15	−1	+1	+1	+1	−1	+1	8.947	15.26	39.80
16	0	0	0	0	0	0	23.53	7.48	26.98
17	0	0	0	0	0	0	23.53	7.48	26.98
18	+1	+1	−1	+1	+1	−1	37.19	10.43	54.77
19	+1	−1	+1	+1	+1	−1	13.99	2.00	14.73
20	0	0	0	0	0	0	23.53	7.48	26.98
21	0	+1	0	0	0	0	28.14	12.47	36.46
22	−1	+1	−1	−1	+1	−1	29.33	10.43	62.50
23	+1	+1	+1	−1	+1	−1	42.85	10.04	37.94
24	−1	+1	−1	+1	+1	+1	17.70	10.43	43.00
25	0	0	0	0	+1	0	19.91	6.17	23.28
26	+1	−1	−1	+1	+1	+1	21.29	2.08	19.20
27	−1	−1	+1	−1	−1	+1	13.44	3.05	19.92
28	−1	−1	−1	+1	+1	−1	8.69	2.08	21.57
29	−1	−1	+1	−1	+1	−1	10.52	2.00	15.64
30	0	0	0	0	−1	0	28.53	9.55	32.24
31	0	−1	0	0	0	0	17.71	2.49	17.76
32	0	0	−1	0	0	0	35.16	7.58	41.99
33	0	0	0	0	0	0	23.53	7.48	26.98
34	+1	+1	−1	+1	−1	+1	54.26	16.19	59.22
35	−1	+1	+1	+1	+1	−1	10.99	10.04	34.95
36	+1	+1	+1	+1	−1	−1	35.28	15.26	45.84
37	−1	+1	−1	+1	−1	−1	24.31	16.19	73.20
38	0	0	0	0	0	0	23.53	7.48	26.98
39	0	0	0	0	0	+1	20.69	7.48	24.76
40	+1	0	0	0	0	0	33.10	7.48	26.73
41	−1	+1	+1	−1	−1	−1	27.01	15.26	51.00
42	0	0	+1	0	0	0	23.51	7.23	27.66
43	0	0	0	+1	0	0	19.40	7.48	25.90
44	+1	−1	+1	−1	+1	+1	20.43	2.00	13.54
45	0	0	0	0	0	−1	22.68	7.48	31.00
46	+1	+1	+1	+1	+1	+1	28.30	10.04	28.34
47	+1	−1	−1	−1	+1	−1	33.47	2.08	23.22
48	0	0	0	0	0	0	23.53	7.48	26.98
49	−1	+1	−1	−1	−1	+1	32.40	16.19	61.90
50	+1	+1	−1	−1	−1	−1	79.55	16.19	82.35
51	−1	−1	+1	+1	−1	−1	9.58	3.05	21.40
52	−1	0	0	0	0	0	14.88	7.48	26.90
53	−1	−1	−1	+1	−1	+1	14.15	3.23	27.73

Table S3. Regression analysis results for the response surface models developed for three output responses deflection angle (Y_1), input power (Y_2), and micromirror plate temperature rise from the ambient (Y_3).

Source	DF	Sum of Squares			Mean Square			*F*-Value			*p*-value (Prob > *F*)		
		Y_1	Y_2	Y_3	Y_1	Y_2	Y_3	Y_1	Y_2	Y_3	Y_1	Y_2	Y_3
**Model**	27	8616.54	1047.14	11446.92	319.13	38.78	423.96	102.90	5526.83	138.90	<0.0001	<0.0001	<0.0001
X_1	1	3307.59	0.000	1.04	3307.59	0.000	1.04	1066.51	0.000	0.34	<0.0001	1.0000	0.5655
X_2	1	1854.95	912.81	6332.26	1854.95	912.81	6332.26	598.11	1.301×10^5	2074.54	<0.0001	<0.0001	<0.0001
X_3	1	738.52	1.31	1914.02	738.52	1.31	1914.02	238.13	186.30	627.06	<0.0001	<0.0001	<0.0001
X_4	1	931.21	0.000	59.70	931.21	0.000	59.70	300.26	0.000	19.56	<0.0001	1.0000	0.0002
X_5	1	726.61	92.43	1079.62	726.61	92.43	1079.62	234.29	13171.36	353.70	<0.0001	<0.0001	<0.0001
X_6	1	35.27	0.000	465.59	35.27	0.000	465.59	11.37	0.000	152.53	0.0024	1.0000	<0.0001
X_1X_2	1	258.05	0.000	0.37	258.05	0.000	0.37	83.21	0.000	0.12	<0.0001	1.0000	0.7305
X_1X_3	1	48.90	0.000	6.933×10^{-3}	48.90	0.000	6.933×10^{-3}	15.77	0.000	2.271×10^{-3}	0.0005	1.0000	0.9624
X_1X_4	1	104.74	0.000	3.140×10^{-3}	104.74	0.000	3.140×10^{-3}	33.77	0.000	1.029×10^{-3}	<0.0001	1.0000	0.9747
X_1X_5	1	51.72	0.000	0.021	51.72	0.000	0.021	16.68	0.000	7.036×10^{-3}	0.0004	1.0000	0.9338
X_1X_6	1	0.073	0.000	0.82	0.073	0.000	0.82	0.023	0.000	0.27	0.8796	1.0000	0.6088
X_2X_3	1	81.20	0.56	358.51	81.20	0.56	358.51	26.18	80.21	117.45	<0.0001	<0.0001	<0.0001
X_2X_4	1	34.04	0.000	15.65	34.04	0.000	15.65	10.97	0.000	5.13	0.0028	1.0000	0.0325
X_2X_5	1	24.72	38.59	85.97	24.72	38.59	85.97	7.97	5499.05	28.16	0.0092	<0.0001	<0.0001
X_2X_6	1	1.89	0.000	147.68	1.89	0.000	147.68	0.61	0.000	48.38	0.4420	1.0000	<0.0001
X_3X_4	1	30.00	0.000	8.98	30.00	0.000	8.98	9.67	0.000	2.94	0.0046	1.0000	0.0987
X_3X_5	1	14.51	0.20	46.65	14.51	0.20	46.65	4.68	29.09	15.28	0.0403	<0.0001	0.0006
X_3X_6	1	2.73	0.000	55.47	2.73	0.000	55.47	0.88	0.000	18.17	0.3572	1.0000	0.0003
X_4X_5	1	25.56	0.000	1.89	25.56	0.000	1.89	8.24	0.000	0.62	0.0082	1.0000	0.4392
X_4X_6	1	140.60	0.000	18.95	140.60	0.000	18.95	45.34	0.000	6.21	<0.0001	1.0000	0.0197
X_5X_6	1	0.25	0.000	4.64	0.25	0.000	4.64	0.080	0.000	1.52	0.7797	1.0000	0.2289

X_1^2	1	0.32	2.079×10^{-7}	0.25	0.32	2.079×10^{-7}	0.25	0.10	2.963×10^{-5}	0.083	0.7512	0.9957	0.7754
X_2^2	1	5.48	9.995×10^{-8}	2.197×10^{-3}	5.48	9.995×10^{-8}	2.197×10^{-3}	1.77	1.424×10^{-5}	7.198×10^{-4}	0.1957	0.9970	0.9788
X_3^2	1	77.86	0.013	140.80	77.86	0.013	140.80	25.11	1.90	46.13	<0.0001	0.1801	0.0001
X_4^2	1	0.19	2.079×10^{-7}	0.12	0.19	2.079×10^{-7}	0.12	0.060	2.963×10^{-5}	0.040	0.8090	0.9957	0.8430
X_5^2	1	0.86	0.34	0.91	0.86	0.34	0.91	0.28	49.00	0.30	0.6040	<0.0001	0.5893
X_6^2	1	8.98	2.079×10^{-7}	1.30	8.98	2.079×10^{-7}	1.30	2.90	2.963×10^{-5}	0.43	0.1012	0.9957	0.5198
**Residual**	25	77.53	0.18	76.31	3.10	7.017×10^{-3}	3.05	-	-	-	-	-	-
**Total**	52	8694.1	-	-	-	-	-	-	-	-	-	-	-
